# The effects of GPER on age-associated memory impairment induced by decreased estrogen levels

**DOI:** 10.3389/fmolb.2023.1097018

**Published:** 2023-03-20

**Authors:** Wenyu Luo, Yudie Yan, Yunpeng Cao, Yanbo Zhang, Zhen Zhang

**Affiliations:** ^1^ Department of Ultrasound, The First Hospital of China Medical University, Shenyang, Liaoning, China; ^2^ Department of Neurology, The First Hospital of China Medical University, Shenyang, Liaoning, China; ^3^ Department of Psychiatry, Faculty of Medicine and Dentistry, University of Alberta, Edmonton, AB, Canada

**Keywords:** G protein-coupled estrogen receptor, estrogen, aging, memory impairment, neurogenesis, synaptic plasticity

## Abstract

Estrogen, as a pleiotropic endocrine hormone, not only regulates the physiological functions of peripheral tissues but also exerts vital neuroregulatory effects in the central nervous system (CNS), such as the development of neurons and the formation of neural network connections, wherein rapid estrogen-mediated reactions positively stimulate spinogenesis and regulate synaptic plasticity and synaptic transmission to facilitate cognitive and memory performance. These fast non-genomic effects can be initiated by membrane-bound estrogen receptors (ERs), three best known of which are ERα, ERβ, and G protein-coupled estrogen receptor (GPER). To date, the effects of ERα and ERβ have been well studied in age-associated memory impairment, whereas there is still a lack of attention to the role of GPER in age-associated memory impairment, and there are still disputes about whether GPER indeed functions as an ER to enhance learning and memory. In this review, we provide a systematic overview of the role of GPER in age-associated memory impairment based on its expression, distribution, and signaling pathways, which might bring some inspiration for translational drugs targeting GPER for age-related diseases and update knowledge on the role of estrogen and its receptor system in the brain.

## 1 Introduction

A dramatic decrease in estrogen synthesis and secretion during perimenopause in women is one of the characteristics of aging, which directly affects memory and cognitive function (van den Beld et al., 2018). Within the brain, estradiol (E2), the most potent and active form of estrogen, is well positioned on brain development, sex differentiation, reproductive behavior, and cognition (including learning and memory) (Vickers et al., 2016; Adhya et al., 2018). Preclinical evidence on the neuroprotective effects of 17β-E2 has also linked menopause to cognitive aging, with nearly twice as many women as men experiencing cognitive decline (Jett et al., 2022). E2 in the hippocampus, derived from the circulatory system and local synthesis, can promote hippocampal neurogenesis (Sager et al., 2018). E2 is also capable of regulating synaptic plasticity in both male and female rats, positively affecting hippocampus-dependent memory (Tuscher et al., 2016; Zhao et al., 2018). Both nuclear- and membrane-associated ERs collaborate in mediating estrogenic actions within the CNS (Lai et al., 2017b).

As classical nuclear ERs, ERα and ERβ act as transcription factors *via* genomic pathways in the nuclear compartment of cells, which interact with nuclear estrogen response elements (EREs) and initiate transcription (O’Malley and Means, 1974). The identification of more ERs increases the complexity of estrogen-mediated signaling pathway network (Jacquot et al., 2021a). In 1990s, an orphan G protein-coupled receptor (GPCR) was named G protein-coupled receptor 30 (GPR30), and later in 2007, the International Union of Basic and Clinical Pharmacology assigned GPR30 as GPER, in concert with the observation of a high affinity for E2 (Thomas et al., 2005; Barton et al., 2018). The first credible genetic case for GPR30 as a novel ER was that rapid estrogen responses can be reproduced in non-responsive breast cancer cells *via* transfection with a GPR30 expression vector (Filardo et al., 2002). Among ERs, GPER seems unconventional, as it shares homology with the family of class A (rhodopsin) GPCRs, such as the angiotensin II 1A receptor and the interleukin 8A receptor (with 28% sequence similarity) (Carmeci et al., 1997; Prossnitz and Arterburn, 2015).

Given that GPER is capable of mediating estrogenic effects, most previous studies on GPER have focused on metabolic disorders, cancer, and cardiovascular function (Jacquot et al., 2021b). Thereafter, a notion is put forward that the effects of GPER can extend far beyond the gonads to the CNS, and experimental results have identified its neuroregulatory role in neurological disorders (Engler-Chiurazzi et al., 2017). Nevertheless, the ligands of GPER still need further investigated (Khan et al., 2022), several specific agonists [G1 (Bologa et al., 2006), CITFA (DeLeon et al., 2022), etc.] and antagonists [G15 (Dennis et al., 2009), G36 (Dennis et al., 2011), etc.] of GPER have been synthesized and characterized. The available application of these selective pharmacological tools in combination with GPER knockout mice may be of great importance to discern responses specific to ERs or GPER and unveil the functions of GPER (Prossnitz and Arterburn, 2015).

However, the investigation of GPER in the neuronal development and memory function is still relatively limited, and the understanding of its effects on age-associated memory impairment needs to be constantly updated. Thus, this review provides a profile of advances about GPER, including its expression, distribution, signaling pathways, and effects from the aspects of neurogenesis, synaptic plasticity and synaptic transmission.

## 2 Expression in memory-related tissues and subcellular distribution of GPER

GPER is ubiquitously expressed throughout the body (Prossnitz and Barton, 2011). In addition to peripheral tissues, such as the placenta, reproductive organs, heart, pancreas, and lung (Prossnitz and Barton, 2011), GPER is highly expressed in the CNS (Cooke et al., 2017), including the hippocampus, cortex, hypothalamus, striatum, and amygdala (Brailoiu et al., 2007; Llorente et al., 2020; Marraudino et al., 2021). Recent transcriptomic analysis of adult rats has shown that GPER transcripts are dominant in the brain compared to peripheral tissues and exhibit higher expression levels compared to classical ERs (Hutson et al., 2019).

Memory impairment induced by normal age or age-related diseases is the primary neurodegenerative symptom leading to biochemical changes in some brain regions, an imperative structure among which is the hippocampus (Wang et al., 2020). And the hippocampus is a brain region that is extremely sensitive to E2 (Uhl et al., 2022). Immunohistochemical studies in the rodent brain show that GPER is colocalized with pyramidal cells in every region of the hippocampus (CA1-3) and granule cells in the dentate gyrus (DG) and is especially dense in the stratum lucidum of CA3 (Waters et al., 2015; Mahmoud et al., 2016). The expression of GPER is influenced by sex and the estrous cycle, which is particularly evident in neurons of the CA1 and DG (Waters et al., 2015). In hippocampal CA1 neurons, GPER immunoreactivity is associated with dendrites, dendritic spines, and axons and is specifically localized to synaptic structures (Akama et al., 2013; Waters et al., 2015). Dual-fluorescent labeling studies of cortical neurons have also shown that GPER is expressed at synapses (Srivastava and Evans, 2013).

Regarding the subcellular distribution of GPER, some researchers have declared that it is expressed in the cell membrane (Thomas et al., 2005; Akama et al., 2013), while there is also evidence that GPER can be localized in the endoplasmic reticulum (Revankar et al., 2005; Camilletti et al., 2019), Golgi complex (Cheng et al., 2011b), and even the nucleus (Pupo et al., 2013). Thus far, the exact location of GPER is still unclear due to the exit of endocytic transport (Cheng et al., 2011a). Rapid endocytosis, rapid degradation, and slow transport experienced by GPER inside the cell may potentially explain the difficulty in detecting GPER on the cell surface (Cheng et al., 2011b). Additionally, the duration of GPER in the plasma membrane in different cell types may mainly depend on the extent of interaction with multiple scaffolding proteins, accessory proteins, and other GPCRs in the plasma membrane (West and Hanyaloglu, 2015). For example, the scaffolding protein PSD-95 can specifically bind to GPER at the postsynaptic density (PSD) region in dendritic spines and prevent its constitutive endocytosis, which extends the retention time of GPER in the membrane and increases its chance to interact with other GPCRs (Akama et al., 2013).

## 3 Signaling pathways of GPER

Despite the genomic and nongenomic responses initiated by estrogen almost ascribed to ERα and ERβ previously, accumulating evidence supports that GPER is capable of eliciting both rapid and transcriptional cellular events (Alexander et al., 2017; Feldman and Limbird, 2017). The non-genomic estrogenic responses encompass rapid activation of ion channels and intracellular second messenger signaling pathways, which occur in a time frame of seconds to minutes (Fuentes and Silveyra, 2019).

After GPER binding to estrogen or estrogen-like molecules, the heterotrimeric G protein splits into Gα and Gβγ subunits and further triggers multiplex intracellular signaling cascades that take part in systemic responses (Barton et al., 2018). Gαs accounts for estrogen-initiated activation of adenylyl cyclase (AC) and the ensuing increase in cAMP generation and protein kinase A (PKA) activity (Thomas et al., 2005). This process also results in intracellular calcium mobilization (Revankar et al., 2005). Gβγ recruits steroid receptor coactivator (SRC) to promote the activation of metalloproteinases (MMPs) and the subsequent cleaving of pro-heparan-bound epidermal growth factor (pro-HB-EGF), releasing HB-EGF from the cell surface, which transactivates EGF receptor (EGFR) (Prossnitz et al., 2008). EGFR activation is accompanied by the activation of extracellular signal-regulated kinase 1/2 (ERK1/2) and phosphoinositide 3-kinase (PI3K) (Filardo et al., 2000; Bustamante-Barrientos et al., 2021). The cAMP-dependent signaling pathway has been shown to negatively regulate EGFR-induced ERK1/2 activity (Filardo, 2002). The details of phospholipase C (PLC) involvement need further investigation (Prossnitz et al., 2008; Lu and Herndon, 2017; Roque et al., 2019).

In the SRC/EGFR/mitogen-activated protein kinase (MAPK) pathway, when GPER couples to E2, phosphorylated c-Jun N-terminal kinase (JNK) regulates actin polymerization to promote dendritic spine formation and memory consolidation in the hippocampal CA1 region, which highlights the importance of actin polymerization in GPER-regulated hippocampal functions in female mice (Kim et al., 2019). On the other hand, ERK, another subfamily of MAPK, not only participates in the inhibitory effect of GPER on the neuronal stem/progenitor cells (NSPCs) proliferation (Zhong et al., 2019) but also activates and enhances synaptic transmission in the hippocampus (Kumar et al., 2015).

In addition, in the PI3K/Akt pathway, p-Akt inhibits GSK-3 to reduce neuronal loss and alleviate cognitive function (Wang et al., 2017). Activation of PI3K/Akt pathway by GPER is associated with the neuritogenesis in developing hippocampal neurons (Ruiz-Palmero et al., 2013). This pathway also engages in the process of axonal myelination and remyelination, which plays a neuroprotective role (Xiao et al., 2018). GPER can signal through PI3K/Akt/mammalian target of rapamycin (mTOR) pathway to reduce glutamate-induced excessive neuronal autophagy and protect neurons from excitotoxicity (Yue et al., 2019). Rapamycin complex 2 (mTORC2), an downstream target of SRC/EGFR/PI3K pathway, is capable of participating in actin cytoskeleton polymerization and long-term memory consolidation (Xing et al., 2018; Zhang Y. Y et al., 2019). Besides, GPER can downregulate ubiquitin-conjugating enzyme 9 (ubc9) expression, which is associated with synaptic plasticity, *via* E2 in cortical neurons through the PI3K pathway (Lai et al., 2017a).

Although GPER is well known for its nongenomic effects, it can mediate transcriptional events as well, which take place in the presence of kinase activation (Prossnitz et al., 2008; Feldman and Limbird, 2017). cAMP response element binding protein (CREB) and c-fos, representative transcription factors, have been implicated in several studies (Cheong et al., 2012). GPER activation can phosphorylate PKA and later stimulate CREB, contributing to learning and memory improvement and long-term memory consolidation in ovariectomized (OVX) rats (Lee et al., 2012; Koss et al., 2018; Machado et al., 2019). GPER-activated CREB can provoke acetylcholine (Ach) release (Filardo and Thomas, 2005; Liu et al., 2019). Phosphorylated CREB *via* cAMP/PKA pathway may also upregulate Bcl-2 expression and avoid oxidative stress-induced apoptosis, which is supposed to be mediated by GPER (Kanda and Watanabe, 2003). Transcriptional activation of c-fos occurs in the downstream of GPER-mediated EGFR/ERK1/2 pathway (Cirillo et al., 2019). As mentioned above, PI3K/Akt pathway can inhibits GSK-3 and ensuingly activate Nrf2, which transfers into the nucleus and combines with transcriptional factors to initiate the transcription of antioxidant enzymes (Wang et al., 2017). In addition, the GPER-initiated PI3K/Akt pathway can also phosphorylate intracellular ERα/β, denoting the beginning of the genomic pathway (Bustamante-Barrientos et al., 2021).

## 4 The effects of GPER on memory impairment

### 4.1 GPER affects memory and cognition by engaging in neurogenesis and reducing neuronal loss

Hippocampal neurogenesis plays an integral role in synaptic plasticity and function, and its perturbation can result in learning and memory impairment (Hollands et al., 2016; Kempermann, 2019). Adult hippocampal neurogenesis declines with age, as has been well established in rodents (Demars et al., 2013) and humans (Khan et al., 2015). Given that newborn neurons are capable of maintaining hippocampal functions and have a positive impact on spatial memory (Kathner-Schaffert et al., 2019), E2 can counteract age-associated memory impairment by promoting neurogenesis in the hippocampus (Sager et al., 2018; Sahab-Negah et al., 2020). In contrast, the degraded regeneration capacity of compromised neurons in mice contributes to long-term cognitive deficits (Kathner-Schaffert et al., 2019).

GPER is universally expressed in NSPCs (Zhang L. et al., 2019; Zhong et al., 2019). Activation of GPER *via* the specific agonist G1 dose-dependently suppresses the proliferation of mouse-derived NSPCs, and this inhibitory effect can be reversed by G15 (Zhong et al., 2019). However, a contradictory finding shows that long-term administration of G1 can promote neurogenesis and ameliorate neuronal damage in CA1 region of the hippocampus in OVX rats, improving spatial memory after neuronal injury (Wang et al., 2021). To investigate the effects of GPER on the estrogen-mediated proliferation of NSPCs, E2 conjugated with bovine serum albumin (E2-BSA) is used to prevent E2 from entering the cell, leading to the conclusion that GPER indirectly engages in the proliferation of NSPCs (Okada et al., 2010). In addition, the region-specific effect of GPER on neurogenesis has been explored, and the results showed that G1 reduces the proliferation of neurons and downregulates the expression of GPER in the DG of adult female rats, whereas G15 increases the number of proliferating neurons in the dorsal DG and decreases their number in the ventral DG (Duarte-Guterman et al., 2015). G15 in combination with E2 only partially counteracts the pro-proliferative effect of E2, indicating that the effect of GPER on neurogenesis is not completely dependent on E2 (Duarte-Guterman et al., 2015). Additionally, GPER does not colocalize with progenitor cells in the subgranular zone of the DG, revealing that the effects of GPER on neurogenesis may be indirect (Duarte-Guterman et al., 2015).

However, accumulating data have shown that GPER can inhibit apoptosis, neuroinflammation, neurotoxicity, and other detrimental factors that result in neuronal damage and degeneration to reduce neuronal loss and improve cognitive function (Huang et al., 2017; Sarchielli et al., 2020). *In vitro* studies have shown that activated GPER can upregulate antiapoptotic proteins such as Bcl-2 and downregulate apoptotic proteins in neurons by stimulating ERs, thereby alleviating neuronal degeneration (Zhou et al., 2018). Cholinergic neurons regulate hippocampal and neocortical learning and memory circuits, and GPER can respond to estrogen to attenuate inflammation in brain cholinergic neurons, contributing to the improvement of cognitive deficits (Sarchielli et al., 2020).

The long-term impact of ischemic injury in hippocampal neurons is a decrease in neurogenesis in the DG, accompanied by a degradation in learning and relearning ability and memory function (Kathner-Schaffert et al., 2019). However, short-term increased neurons after stroke exhibit the characteristics of abnormal basal dendrite morphology and mislocalization to the hilus of the DG. They either integrate into the established hippocampal circuits but have a shortened lifespan or fail to integrate correctly into the preexisting network, both of which can potentially result in hippocampus-dependent memory impairment (Niv et al., 2012; Woitke et al., 2017). The declining expression of GPER mRNA and protein in the hippocampal CA1 region after stroke reduces the responsiveness of neurons to E2, but E2 treatment immediately after ovariectomy can restore GPER mRNA and protein levels and alleviate ischemic-induced hippocampal neuronal loss, suggesting that GPER mediates the protective effects of E2 on neurons and reduces neuronal loss after neuronal injury (Wu et al., 2018). Single-onset traumatic brain injury is also associated with acute neuronal loss in the hippocampus, and G1 can ameliorate early-onset cognitive deficits and neuronal death by activating the PI3K/Akt pathway (Wang et al., 2017). G1 also significantly mitigates Aβ1-42-induced neuronal toxicity (Kurt et al., 2019) and alleviates the impairment of novel object recognition memory in female 5XFAD mice (Kubota et al., 2016).

Although there still exist some contradictions in the role of GPER in hippocampal neurogenesis, the fact cannot be excluded that GPER indeed participates in the protective process of damaged neurons, elongating neuronal survival to reduce neuronal loss and further improving cognitive function after neuronal injury.

### 4.2 GPER affects memory and cognition by regulating synaptic plasticity

Remodeling of synaptic structure and upkeep of synaptic function are essential to the cognitive function (Srivastava et al., 2013). GPER is enriched at synapses in the brain and is involved in the rapid regulation of hippocampal dendritic morphology and synaptic plasticity (Alexander et al., 2017). G1 treatment can increase dendritic spine density in the hippocampal CA1 region in a non-genomic way (Gabor et al., 2015). The newly identified GPER-specific agonist CITFA can significantly promote axonal and dendritic growth in hippocampal neurons of E18 fetal rats (DeLeon et al., 2022). In addition, overexpression of GPER in cortical neurons leads to an increase in spine synaptic density (Srivastava and Evans, 2013). Further research performed in monkeys shows that a higher expression of GPER adjacent to the PSD corresponds to a higher synaptic density of dendritic spines in this region, revealing the critical role of GPER in synaptic plasticity (Crimins et al., 2017). The increased dendritic spine density in CA1 may be strongly linked to the facilitation of learning and memory by GPER (Gabor et al., 2015). Actin polymerization underlies GPER-mediated dendritic spine generation and memory consolidation in the hippocampal CA1 region (Kim et al., 2019). GPER is reported to regulate learning and memory through the ERα/ERβ/SRC and PI3K/Akt pathways, followed by activation of mTORC2 and downstream actin polymerization to govern synaptic protein expression, suggesting that GPER can modulate structural plasticity in the hippocampal CA1 region (Zhang Y. Y et al., 2019; Li et al., 2021). mTORC2 activation can remarkably rescue the G15-induced decrease in dendritic spine density and spatial memory disorder (Xing et al., 2018). GPER activation can also increase dendritic spine density and enhance memory consolidation through a pathway where JNK phosphorylation and cofilin phosphorylation contribute to actin polymerization (Kim et al., 2016; Kim et al., 2019) but not through E2-mediated ERK phosphorylation (Kim, 2014). Intriguingly, in the developing hippocampus, GPER activation markedly increases the density of dendritic spines and alters the expression of related synaptic proteins in the stratum lacunosum moleculare (SLM) of the hippocampal CA1 in female rats (Li et al., 2021). However, these increased dendritic spines do not translate into increased synaptic density, implying that GPER activation only modifies the structural plasticity of the developing hippocampal CA1 region, and this effect is gender-specific (Li et al., 2021).

Dendritic spines are specialized projections of neurons, and alterations in dendritic spine function directly affect synaptic plasticity (Dorostkar et al., 2015). Memory is encoded by changing synaptic strength through long-term potentiation (LTP) and long-term depression (LTD) (Nabavi et al., 2014). GPER-mediated mGluR-LTD in CA3 represents a novel estrogen-mediated regulation of synaptic plasticity (Briz et al., 2015). GPER activation by G1 induces a rapid release of brain-derived neurotrophic factor (BDNF) that transiently stimulates the translation of activity-regulated cytoskeleton-associated protein (Arc) and internalization of GluA1-containing AMPA receptor in the CA3 region (Briz et al., 2015). However, unlike the induction of mGluR-LTD *via* E2 in CA1, mGluR activation in CA3 does not increase the synthesis of Arc; instead, it promotes the internalization of GluA1 under the premise of GPER activation and causes GluA1 to be rapidly degraded by the ubiquitin-proteasome system, triggering LTD (Briz et al., 2015). That GPER activation enhances mGluR-LTD in mossy fiber-CA3 synapses also explains the improving effects of G1 on contextual and spatial memory in middle-aged mice (Xu et al., 2018). LTD plays a role in regulating the transcription, translation, and recycling of receptors, which results in the downregulation of cellular excitability and affects learning and memory (Mahmoud et al., 2016). Thus, GPER-induced mGluR-LTD in CA3 lends support to the modulation of synaptic functional plasticity *via* GPER in the hippocampus.

Ubc9 plays an indispensable role in synaptic functional plasticity (Lai et al., 2017a). GPER can reduce the interaction of ubc9 with the postsynaptic protein PSD-95 and increase its interaction with the presynaptic protein synaptophysin *via* the PI3K pathway, and the accumulation of presynaptic ubc9 may inhibit synaptic transmission (Lai et al., 2017a). G1 can increase the expression of PSD-95 in the CA3 region of the mouse hippocampus (Waters et al., 2015). Overexpression of PSD-95 promotes the maturation of glutamatergic synapses and enhances the activity of postsynaptic glutamate receptors, indicating that PSD-95 coordinates synaptic development (El-Husseini et al., 2000). PSD-95 also increases the number and size of dendritic spines, suggesting its role in synaptic stability and plasticity (El-Husseini et al., 2000).

### 4.3 GPER affects memory and cognition by enhancing synaptic transmission

The anatomical localization of GPER at synapses suggests that GPER may play a role in regulating neurotransmission (Wang et al., 2007; Akama et al., 2013). By applying 17β-E2-3-benzoate (EB) and agonists and antagonists of ERs to investigate the contribution of each ER, it was found that ERα, ERβ, and GPER are all involved in EB-mediated enhancement of synaptic transmission (Kumar et al., 2015). However, among them, GPER is the leading contributor to the EB-mediated increase in ERK activation and the CA3-CA1 synaptic response in the hippocampus of OVX female mice (Kumar et al., 2015). GPER can also alter the efficacy of excitatory synaptic transmission at Schaffer-collateral-CA1 synapses. Indeed, G1 is reported to mirror the actions of E2 to constantly potentiate excitatory synaptic transmission at CA1 synapses (Smejkalova and Woolley, 2010).

There is a strong connection between cholinergic signaling and cognitive function in the CNS (Ballinger et al., 2016). Levels of choline acetyltransferase (ChAT) and acetylcholinesterase (AchE), major markers of cholinergic neuronal activity, are reduced in the late stage of Alzheimer’s disease, which contributes to cognitive deficits (H. Ferreira-Vieira et al., 2016). GPER is expressed in the majority of cholinergic neurons in the basal forebrain, and GPER activation effectively improves the function of hippocampal cholinergic neurons in OVX rats (Hammond et al., 2011).

Experimental results demonstrate that short-term G1 treatment in OVX rats productively enhances spatial recognition memory relying on environmental novelty, which may be based on the fact that GPER activation increases the release of Ach from the basal forebrain to the hippocampus and that elevated Ach levels in the hippocampus can lead to an increase in synaptic connectivity and interneuron communication during learning (Hawley et al., 2014). Moreover, long-term G1 administration in OVX rats increases hippocampal potassium-stimulated Ach release by 3-fold and improves rat performance in a maze test (Hammond et al., 2011). Thereafter, this team extended their previous conclusion and illustrated that E2 treatment can improve task-specific learning by activating GPER and enhancing Ach release associated with food reward (Gibbs et al., 2014), which suggests that both short-term and long-term G1 treatment can strengthen hippocampal cholinergic neuronal function and improve learning and memory. In contrast, long-term G15 administration impairs the performance in the maze test and slows the rate of spatial memory acquisition, which is similar to the effect of removing cholinergic inputs to the hippocampus and frontal cortex, suggesting that GPER may mediate the effects of E2 on basal forebrain cholinergic function to modulate spatial memory (Hammond et al., 2012). Cholinergic neurons in the basal forebrain also engage in GPER-mediated early consolidation of object recognition memory and emotional memory in adult male rats (de Souza et al., 2021).

Followed by the activation of cAMP and CREB, GPER increases the activity of the AchE promoter in PC12 neurons, while GPER increases miR-132 levels, which is associated with the inhibition of AchE translation (Liu et al., 2019). Thus, GPER may maintain the homeostasis between Ach synthesis and degradation to improve cognitive and memory function in neurodegenerative diseases (Liu et al., 2019).

## 5 Conclusion and perspectives

**FIGURE 1:**
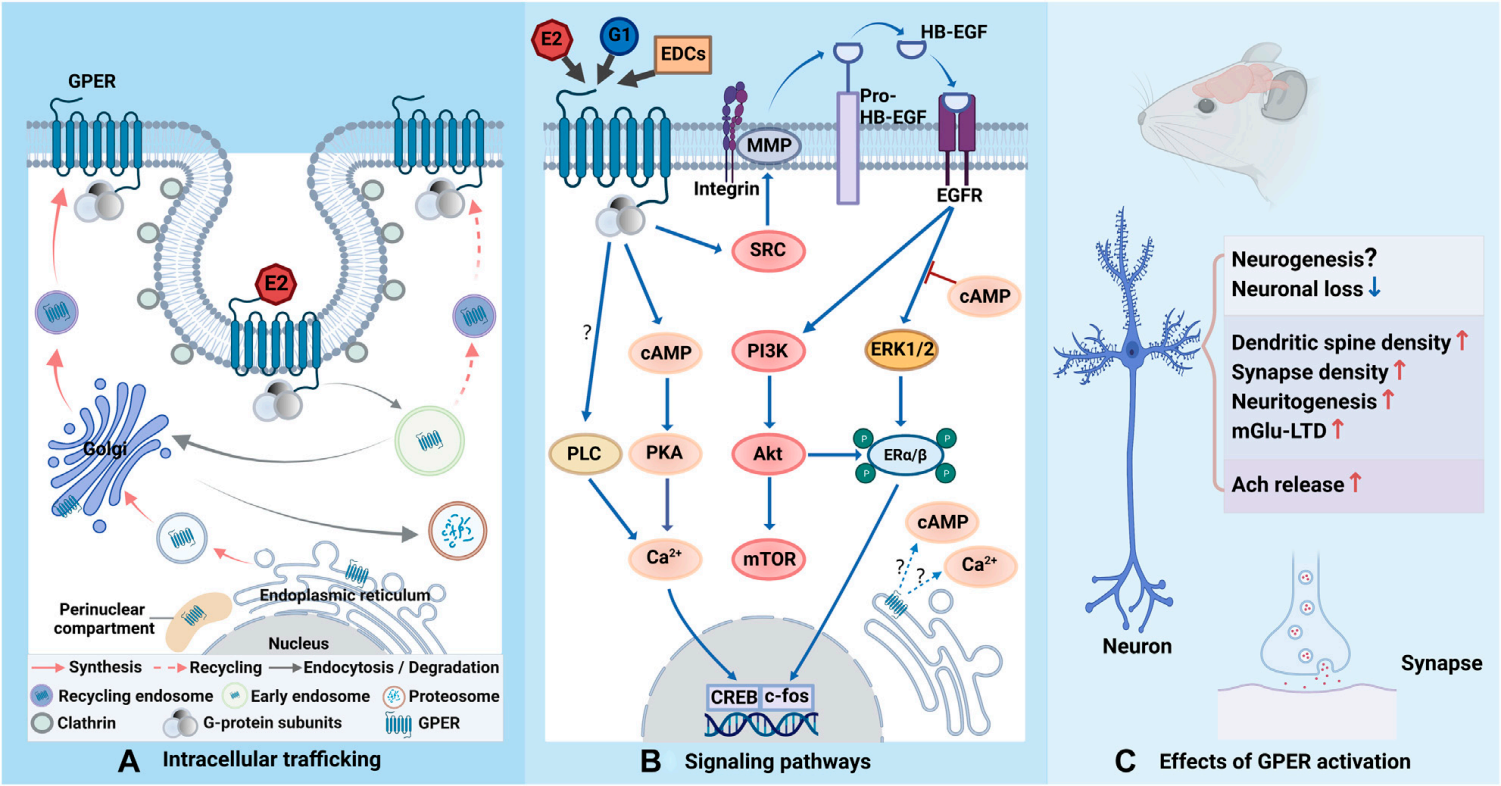
Schematic representation of GPER trafficking, related signaling pathways and effects. **(A)** GPER is biosynthesized in the endoplasmic reticulum and processed in the Golgi apparatus before its trafficking to the plasma membrane. When binding to estrogen or estrogen-like molecules, GPER is constitutively endocytosed into cells *via* clathrin-coated vesicles and enters the early endosome. The majority of GPERs are transported to the Golgi apparatus and degraded *via* the ubiquitin-proteasomal system, while a minority of them are recycled back to the cell membrane. GPER is also reported to accumulate in the perinuclear compartment. **(B)** Upon activation, the G protein departs into Gα and Gβγ subunits and further triggers complicated signaling cascades. Gβγ stimulates SRC-activated MMPs, leading to the release of HB-EGF from the cell surface, which transactivates EGFR and subsequently triggers ERK1/2 and PI3K. Gαs activates AC and increases cAMP generation, which results in intracellular calcium mobilization. cAMP also recruits PKA to activates CREB in the nucleus to initiate transcription, which can also be potentiated by phosphorylated ERα/β *via* ERK and Akt. The cAMP-related signaling pathway can regulate EGFR-induced ERK1/2 activity. It is not yet clear whether GPER signaling can be observed from intracellular receptors. The involvement of PLC may depend on cell type (question marks). **(C)** Activation of GPER can exert neuroprotection to reduce neuronal loss, but the role of GPER in neurogenesis necessities further investigation. Activation of GPER can positively regulate synaptic formation and plasticity. GPER can also mediate E2 effect to increase Ach release. The figure was created with BioRender.com.

In summary, GPER can trigger a rapid estrogenic response to improve hippocampal memory and cognitive function, either by acting on the neurogenesis or by affecting hippocampal synaptic function and the activity of cholinergic input into the hippocampus (Table 1). The emergence of GPER fills the gap in the mechanisms of estrogen-mediated neuroprotection, suggesting that GPER can be applied as a promising target to alleviate aging-induced memory decline and cognitive dysfunction. Since GPER can elicit both non-genomic signaling and transcriptional progress, further studies are needed to clarify the precise subcellular localization of GPER and identify the potential interactions between membrane-associated signaling pathways and nuclear-associated signaling pathways to provide a robust theoretical basis for the therapeutic role of GPER in aging-induced memory impairments. Future studies may also require the discovery or synthesis of more agonists, antagonists, and modulators with higher affinity and specificity for GPER to deeply explore its regulatory mechanisms. Another urgent issue is the lack of a crystal structure for GPER. Furthermore, considering that most studies of estrogen and GPER are performed in cellular models and OVX female rodents, more researches are needed to understand how GPER regulates memory in the human CNS in normal and pathological conditions (Figure 1).

**TABLE 1:** The effects of GPER in memory impairment.

	Estrogen or estrogen-like molecules	Models	Cells/Tissues	Main findings	References
Neurogenesis	E2	E12.5 fetal mice	NSPCs from cortex	GPER expresses in NPSCs and its activation inhibits the proliferation in a dose-dependent manner	Okada et al. (2010)
	G1				Zhang L. et al. (2019)
	G15				Zhong et al. (2019)
	E2	Adult female rats	NSPCs from hippocampus	GPER region-specifically impacts cell proliferation in hippocampal DG. G1 reduces the proliferation of neurons, whereas G15 increases the number of proliferating neurons in the dorsal DG and decreases the number of them in the ventral DG	Duarte-Guterman et al. (2015)
	G1				
	G15				
	E2	OVX rats with GCI	NSC in the hippocampal SGZ and CA1 regions	Long-term GPER activation promotes neurogenesis	Wang et al. (2021)
	G1				
Neuroprotection	Raloxifene	ALS	TDP-25 cells	Raloxifene provides neuroprotection through GPER by increasing autophagy, decreasing apoptosis and enhancing cell viability	Zhou et al. (2018)
	E2				
	G1				
	G15				
	E2	Neuroinflammation	primary hfNBM	GPER activation attenuates inflammation in brain cholinergic neurons and improves cognitive deficits	Sarchielli et al. (2020)
	G1				
	G15				
	E2	Male rats after TBI	Hippocampal neurons	GPER activation can improve early-onset cognitive deficits and neuronal death	Wang et al. (2017)
	G1				
	E2	OVX rats with GCI	Hippocampal neurons	E2 has a therapeutic window to exert neuroprotection and declining expression of GPER reduces the responsiveness of neurons to E2	Wu et al. (2018)
	G1				
	G15				
	G1	5XFAD mice	Primary rat cortex neurons	GPER activation markedly attenuates Aβ1-42-induced neuronal toxicity and mitigates impairment of novel object recognition memory in female 5XFAD mice	Kubota et al. (2016)
	G15				Kurt et al. (2019)
Synaptic plasticity	G1	OVX female mice	Hippocampal neurons	GPER-induced rapid increase of dendritic spine density in CA1 correlates with the facilitation of learning and memory	Gabor et al. (2015)
	CITFA	E18 fetal rats	Hippocampal neurons	CITFA can significantly promote axonal and dendritic growth	DeLeon et al. (2022)
	E2	OVX rhesus monkeys	Neurons from the DPC	The higher expression levels of GPER adjacent to PSD means the higher synaptic density in this region	Crimins et al. (2017)
	G1				
	G1	OVX female rats	Hippocampal CA1 neurons	GPER activation increases dendritic spine density and enhances memory consolidation through a pathway in which JNK and cofilin phosphorylation contribute to actin polymerization	Kim et al. (2016)
					Kim et al. (2019)
	G1	OVX female mice	mHippoE-14 from embryonic mouse	GPER regulates learning and memory through ERα/ERβ/SRC and PI3K pathways, followed by activation of mTORC2 and downstream actin polymerization	Zhang Y. Y et al. (2019)
	G15				
	E2	Mice expressing eGFP	Organotypic entorhinal-hippocampal cultures; Hippocampal CA1 neurons	GPER activation only affects the structural plasticity of the developing hippocampal CA1 region, and this effect is gender-specific	Li et al. (2021)
	G1				
	G1	OVX female rats	Hippocampal CA3 neurons	GPER activation induces a rapid release of BDNF and promotes the internalization and degradation of GluA1, triggering LTD.	Briz et al. (2015)
	G1	Middle-aged mice	Hippocampal slices	GPER activation enhances mGluR-LTD in mossy fiber-CA3 synapses and ameliorates fear and spatial memory	Xu et al. (2018)
	G15				
	E2	OVX APP/PS1 mice	Cortical neurons	GPER activation downregulates the mRNA and protein levels of ubc9 and reduces SUMOylation of it	Lai et al. (2017a)
Synaptic transmission	E2	OVX female rats	Hippocampal neurons	Both short-term and long-term G1 treatment can strengthen the function of hippocampal cholinergic neurons and improve learning and memory, while long-term G15 has the opposite effect	Hammond et al. (2011)
	G1				Hammond et al. (2012)
	G15				Gibbs et al. (2014)
					Hawley et al. (2014)
	E2	Adult male rats	**—**	Cholinergic neurons in the basal forebrain may participate in GPER-mediated early consolidation of object recognition memory and emotional memory	de Souza et al. (2021)
	G1				
	G15				

Notes: NSPCs: Neural stem/progenitor cells; GCI: Global cerebral ischemia; DPC: Dorsolateral prefrontal cortex; SGZ: Subgranular zone; OVX: Ovariectomy; hfNBM: Human cholinergic neurons from the fetal nucleus basalis of meynert; TBI: Traumatic brain injury; ALS: Amyotrophic lateral sclerosis; PSD: Postsynaptic density; eGFP: enhanced green fluorescence protein.
